# An approach to automatic classification of *Culicoides* species by learning the wing morphology

**DOI:** 10.1371/journal.pone.0241798

**Published:** 2020-11-04

**Authors:** Pablo Venegas, Noel Pérez, Sonia Zapata, Juan Daniel Mosquera, Denis Augot, José Luis Rojo-Álvarez, Diego Benítez

**Affiliations:** 1 Colegio de Ciencias e Ingenierías “El Politécnico”, Universidad San Francisco de Quito USFQ, Quito, Ecuador; 2 Instituto de Microbiología, Colegio de Ciencias Biológicas y Ambientales “COCIBA”, Universidad San Francisco de Quito USFQ, Quito, Ecuador; 3 Usc Vecpar, ANSES LSA, EA7510, Université de Reims Champagne-Ardenne, Reims, France; 4 Department of Signal Theory and Communications, Rey Juan Carlos University, Fuenlabrada, Spain; Universidad de Granada, SPAIN

## Abstract

Fast and accurate identification of biting midges is crucial in the study of *Culicoides*-borne diseases. In this work, we propose a two-stage method for automatically analyzing *Culicoides* (Diptera: Ceratopogonidae) species. First, an image preprocessing task composed of median and Wiener filters followed by equalization and morphological operations is used to improve the quality of the wing image in order to allow an adequate segmentation of particles of interest. Then, the segmentation of the zones of interest inside the biting midge wing is made using the watershed transform. The proposed method is able to produce optimal feature vectors that help to identify *Culicoides* species. A database containing wing images of *C. obsoletus*, *C. pusillus*, *C. foxi*, and *C. insignis* species was used to test its performance. Feature relevance analysis indicated that the mean of hydraulic radius and eccentricity were relevant for the decision boundary between *C. obsoletus* and *C. pusillus* species. In contrast, the number of particles and the mean of the hydraulic radius was relevant for deciding between *C. foxi* and *C. insignis* species. Meanwhile, for distinguishing among the four species, the number of particles and zones, and the mean of circularity were the most relevant features. The linear discriminant analysis classifier was the best model for the three experimental classification scenarios previously described, achieving averaged areas under the receiver operating characteristic curve of 0.98, 0.90, and 0.96, respectively.

## Introduction

Biting midges of the genus *Culicoides* Latreille, 1809 (Diptera: Ceratopognidae) are hematophagous insects that are widely distributed around the world, and they are known to transmit multiple diseases of veterinary and medical importance [1]. More than 50 arboviruses have been isolated from *Culicoides*, most of them belonging to the *Bunyaviridae* (20 viruses), *Reoviridae* (19 viruses), and *Rhabdoviridae* (11 viruses) [2] familiies. In animals, they are involved in the transmission of bluetongue virus (BTV), Akabane virus (AKAV), bovine ephemeral fever virus (BEFV), epizootic haemorrhagic disease virus (EHDV), Schmallenberg virus (SBV), and african horse sickness virus (AHSV). The circulation of BTV is of special interest in countries like Ecuador, where the presence of these viruses and their possible vectors have only been partially studied [3]. Several species of *Culicoides* also transmit protozoan parasites (*Haemoproteus spp*. and *Leucocytozoon spp*.), and helminth parasites (*Onchocerca*). In humans, *Culicoides paraensis* serve as vectors of the Oropuche virus (OROV) [4]. Infections in humans caused by OROV are characterized as an acute febrile illness with typical clinical symptoms such as fever, headache, muscle and joint pain, or skin rash, which may develop into meningitis and/or encephalitis [5]. *Culicoides paraensis* is one of the most frequent and widespread ceratopogonid midges in the South American continent. The *Culicoides paraensis* group includes six closely related/similar species. (*C. diversus*, *C. filiductus*, *C. neoparaensis*, *C. paraensis*, *C. peruvianus*, and *C. quasiparaensis*). Moreover, the wings pattern of *C. peruvianus* shows a significant intraspecific variation that can even exceed interspecific variation [6].

Entomological surveillance of known vectors species can help in understanding their population structure, distribution, size and means of dispersion. These factors are of great importance in determining *Culicoides* success in transmitting diseases, as well as in establishing control and prevention measures [4]. Therefore, a timely and accurate identification of biting midges is crucial in the study of *Culicoides*-borne diseases. Approximately 1400 species of *Culicoides* have been described and grouped in subgenera (31 or 32), species groups (38 subgenera unplaced), miscellaneaous unplaced species [7, 8], and species complexes [1]. The identification of *Culicoides* at any taxonomic level within the genus relies mainly on morphological and morphometrical parameters, mainly based on features related to the wing [9, 10]. An interactive identification key protocol for *Culicoides* has been previously developed to define the morphological descriptors required for accurate identification [11], where six taxonomists have validated the key, and its success rate ranged from 35.1% to 81.1% depending on *Culicoides* species concerned and users’ levels of expertise in *Culicoides* identification. Despite the results by our group and by others in the field, the current morphometric process for the identification of *Culicoides* relies on manual detection and identification of critical landmarks, which is a laborious process depending on the ability and expertise of a limited number of entomologists, and which can suffer from bias from one expert to another (for instance the number and position of landmarks varied from 10 to 14 in previously reported analysis) [12–16].

To alleviate this problem, several automated approaches have been proposed in the literature for classifying other genera of insects using the analysis of their wing’s morphology, such as the specific amount of points in the wings called landmarks [17], or by studying the wing’s veins and their canonical variate [18]. For example, Lorenz et al. [17] used 32 wing shape features in conjunction with a multilayer perceptron classifier to distinguish among 17 species of the genera *Anopheles*, reaching accuracy rates ranging from 85.70% to 100%. Sontiguna et al. [19] employed wing morphometric analysis features and discriminant function analysis to classify 12 species of flesh flies, achieving accuracy between 81.3% and 100%. Wilke et al. [18] also employed geometric morphometric analysis to classify mosquitoes from three of the most important epidemiologically genera using morphological spaces produced by canonical variate analysis, and their method obtained 96% and 84% accuracy for two different datasets. Yang et al. [20] focused on the wing outline employing elliptic Fourier descriptors and used support vector machines as classifier stage, reaching accuracy ranged from 90% to 98% for different species. Wing pigmentation patterns can be used also in some vector species as the principal criteria for identification, since adults have a remarkable distribution and color of wing spots. Haarlem and Vos [21] employed a conjunction of different algorithms (such as BGR, SURF and Bag-Of-Words) achieving accuracy of about 77% for different species. In general, wing geometric morphometrics is a promising approach to the identification of entomologically relevant insects due to its many advantages regarding accuracy, repeatability, rapidity, and affordability [22]. In the case of *Culicoides*, wing pigmentation patterns could be used in certain species as the principal criteria for identification, since adults also have a remarkable distribution and color of wing spots [12–15].

In this regard, in previous works [23, 24], we have demonstrated that the analysis of wing spots (pale or dark spots shown as particles or bright areas within the wing image) could also be used to differentiate *Culicoides* species by an automated system. The initial study of Guerrón et al. [23] focused primarily on the detection of the particles (bright areas) in the wings. The method proposed therein is based on the use of a Gaussian filter for noise removal, the determination of a threshold to obtain a binary image, and the use of several morphological operations to get the particles within the biting midges wings. An improved version was presented in [24] by Benalcázar et al., where the watershed transformation was added to obtain the number of zones of the wings. Several morphological features as the number of particles, centroid size, biggest particle, elongation, compactness, circularity, hydraulic radius, ellipse ratio, and rectangle radio were also computed from the resulting images. However, in those studies, although we demonstrate that the image processing algorithm improves wing images and that the morphological features obtained have been shown to be different for the *Culicoides* species used as examples (*C. tetrathyris*, *C. glabellus*, and *C. glabrior*), no classification analysis was performed in either study.

In accordance to the previous considerations, the method proposed in this paper is focused on the marks (brighter areas) of the wings. Our current approach has been improved concerning such early versions of the *Culicoides* wing image processing methods, regarding the prepossessing and particle detection steps. The algorithms employed herein increase the quality of the image to detect all the marks within the wing’s images and to preserve their original shape and size. One of the principal advantages is the use of Otsu’s method [25], to determine the threshold to binarize the image. The application of the watershed transformation is also improved by the correct use of masks, to provide therefore better information about the zones in which the wing can be divided, and to have the right information about the wing contour. The use of better algorithms allows us to compute better features and to put them into test with several machine learning classifiers (MLCs).

Hence, in this study we describe a new approach for automated species stratification classification of *Culicoides* based on image processing of wing spots and machine learning techniques. We focus on these techniques to segment the spots in the wing image, which will be subsequently used to form a feature vector containing seven mathematical descriptors per image. An experimental dataset containing wing images from three BTV transmitting species including *C. pusillus* (French Guyana), *C. obsoletus* (France), *C. insignis* (Ecuador), and a non-transmitting species *C. foxi* (Ecuador), was used to benchmark the proposed approach, using five well known MLCs.

## Materials and methods

### Entomological collection

We analyzed a total of 192 wings from females of wild biting midges. Biting midges were collected using CDC-like and UV traps. For the Ecuadorian specimens, traps were set in Cotundo (00°51’05” S, 77°47’65” W), Napo Province, and in Paraiso Escondido (00° 85’ 03” N, 79° 17’ 49” W), Pichincha province. The preparation and mounting of female specimens was carried according to Mosquera [3] and species identification was based on wing patterns described by Spinelli et al. [26]. *C. insignis* was identified in Cotundo and *C. foxi* in Paraiso Escondido. A Zeiss V20 microscope coupled with an AmScope 18MP USB 3.0 color CMOS C-mount microscope camera (magnification X10) was used for obtaining images of the wings. The other biting midges species were captured in French Guyana (3°59′56″ N, 53°00′00″ W) for *C. pusillus*, and in France (49°59’69” N, 4°01’45” E; 43°58’55” N, 3°42’58” E) for *C. obsoletus*. The individuals were stored in 70% ethanol before morphological analysis, specimen identification, and mounting. *Culicoides spp*. were separated from other insects according to their wing characteristics using a stereomicroscope [27] and identified at species level [11, 28]. For these specimens, digital images of the wings were obtained using an Olympus BX53 microscope equipped with an Olympus SC100 camera (magnification X10) with Stream motion software (Olympus).

For our study, left and right wings were used without any distinction because: (i) systematic selection of one side may bias the results in case of differential directional asymmetry among species [29]; (ii) comparison of wings from catalogs and original descriptions with status (left or right) are mostly unknown [16]; (iii) distribution and color spots on both wings are similar [27].

### Database

An image database was assembled, with 192 biting midges wing images comprised of 66 image samples from *C. insignis*, 42 images samples from *C. foxi*, 42 images samples from *C. pusillus*, and 42 image samples from *C. obsoletus*. This database was used for both testing and benchmarking the proposed method.

However, since *C. insignis* and *C. foxi* are two cryptic species (i.e. morphologically identical to each other but belonging to different species), and since *C. pusillus* presents similar morphological characters with *C. obsoletus*, we decided to separate the original database in two image databases, containing similar species. The first one, called *fDatabase* contained 84 wing images from *C. pusillus* (42 images) and *C. obsoletus* (42 images) species, whereas the second one, called *eDatabase*, contained 108 wing sample images from *C. insignis* (66 images) and *C. foxi* (42 image) species. The main idea in this database separation was to test the performance of the proposed method for automated identification and classification when comparing similar species that will be more challenging than comparing species with apparent morphological differences.

### Proposed method

**Fig 1:**
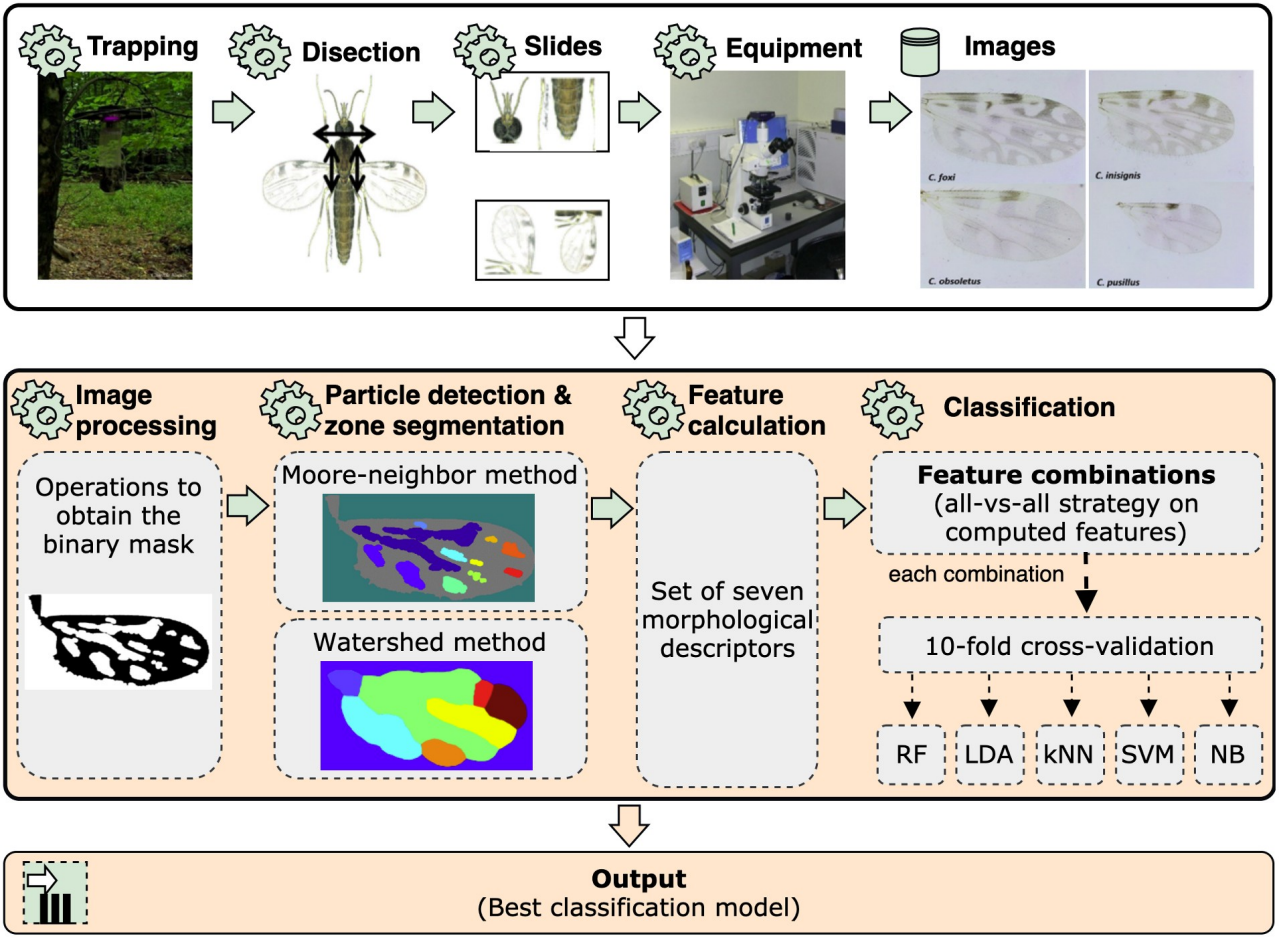
Schematic of the entire research workflow, including the proposed method (central and lower blocks).

The proposed method aims to highlight the main characteristics of the biting midges wings while preserving their details to maximize the species classification. For this purpose, several image processing techniques were used to obtain the binary mask of the wing. Then, the determination of the wing particles and the segmentation of zones provide two images that are used to calculate a set of seven morphological features. The features are then combined using an all-versus-all strategy to form multiple subsets of features that feed a 10-fold cross-validation method along with five MLCs. Finally, the best classification scheme (output) among all models is determined. The workflow of the entire process, including the proposed method, is shown in Fig 1.

#### Image preprocessing

The purpose of the image processing stage is to obtain a binary mask and a bounding box containing the object of interest within the image, which, in our case, is the specimen wing. Towards this goal, the original image was first transformed from the RGB (red, green, blue) color space to a gray space, and therefore redistributing the pixels intensity values in the range from 0 to 255. Then, we used a two-step filtering process with the median and Wiener filters using convolutional masks of (15 × 15) and (25 × 25) respectively, to remove noise while preserving the contour of the objects within the image. Besides, the adaptive histogram equalization method was applied between both filters to improve the contrast of the image by zones. Afterward, an image complement and a morphological dilate operation with a kernel composed of a structuring element type of disk and size 100 on the filtered image was carried out for removing small non-desired objects, such as isolated pixel neighborhoods, veins, or noise captured by the camera that were not removed by the previous step. This morphological operation allowed filling the holes inside the wing, hence providing the final binary mask needed for a subsequent bounding box calculation throughout the use of the Otsu’s method [25], which determines the optimum conversion threshold by minimizing the intra-class variance between two assumed pixel classes. However, preparing the binary mask for detecting the critical particles inside the wing sometimes represents a hard task in image processing due to the strong connections of non-desired objects that overlap the desired ones (in our case, the particles). Thus, the closing and opening morphological mathematics operations with a disk-based structuring element of size 5 and 7, respectively, were also applied to retain the shape and size of key particles without overlapping (see Fig 1, step 1).

#### Wing particles detection and zones segmentation

The wing particle detection is based on the application of the Moore-Neighbor tracing algorithm [30] modified by Jacob’s stopping criteria on the binary wing images (see Fig 1, step 2). This algorithm determines the existence of the particle by analyzing whether or not there are intensity value changes in the neighborhood of the 8-connected pixels of the current pixel (pixel under analysis). Also, it considers any intensity change as the stopping criterion.

**Fig 2:**
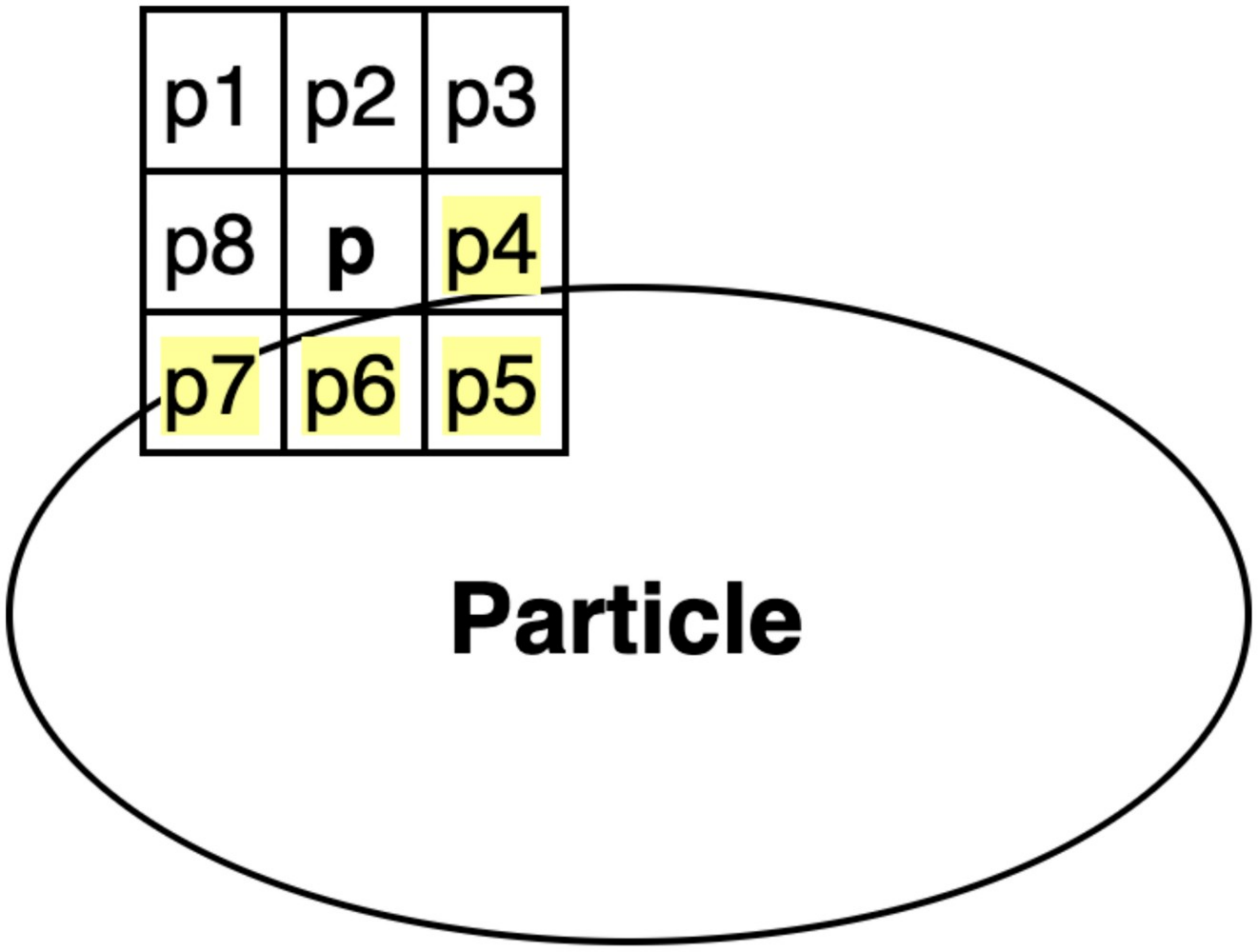
Example of the application of the Moore-neighbor algorithm for particle detection.

That means, the modified Moore-neighborhood of a pixel *p* is a set of pixels {*p*1, *p*2, *p*3, *p*4, *p*5, *p*6, *p*7}, which shares its vertex or edge with the current pixel *p*, as shown in Fig 2. If the center pixel *p* is black, the algorithm will search for a white pixel (pixels of the particle, e.g., the cells highlighted in yellow in Fig 2) in its neighborhood until it achieves success. Otherwise, it will continue the process without stopping.

**Fig 3:**
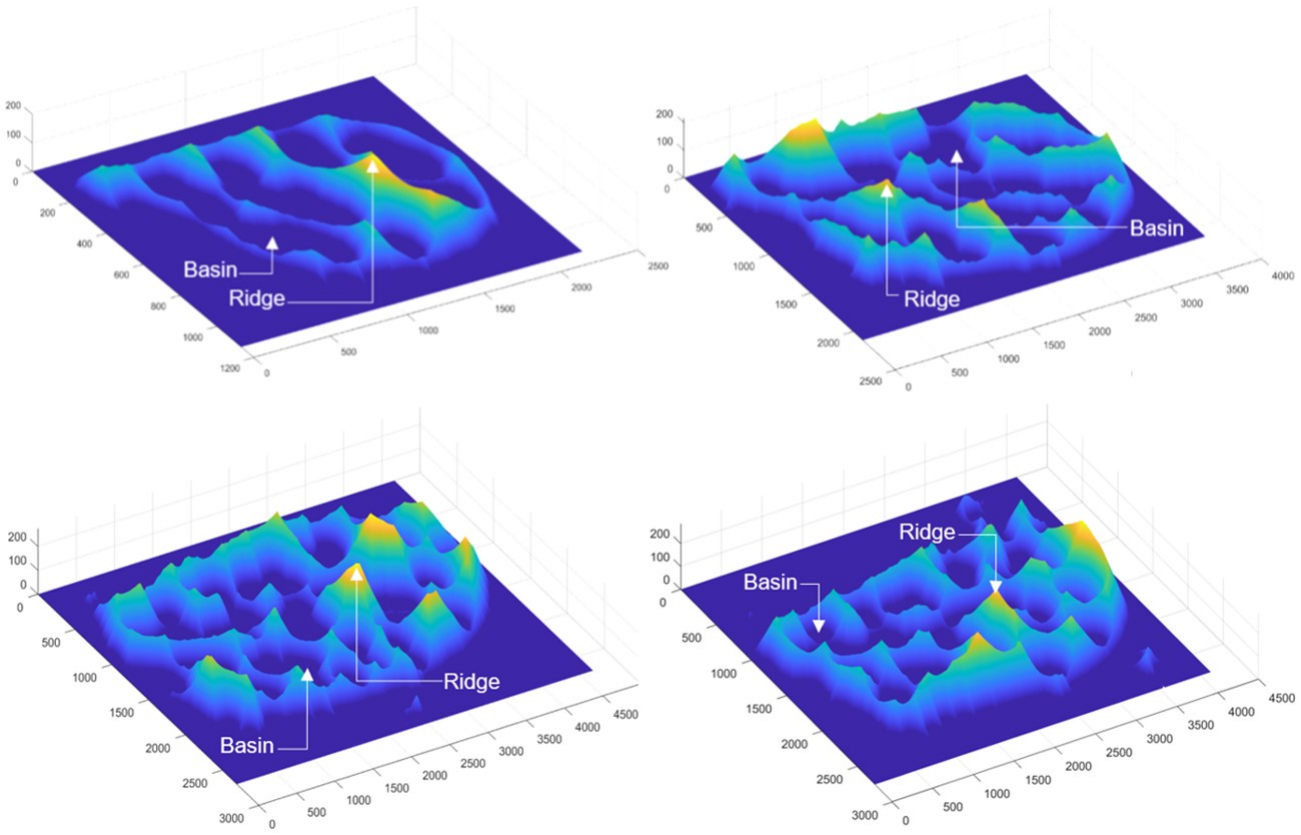
Zone segmentation for *C. pusillus* (top left), *C. obsoletus* (top right), *C. foxi* (bottom left) and *C. insignis* (bottom right).

On the other hand, zones segmentation is based on the application of the watershed transform [31]. This method finds the basins or ridges in the image by treating it as a surface where light and dark pixels represent elevations and depressions, respectively. In this setting, the wing zones are related to the depression region (basins), while the gaps among the wing particles are linked to the elevation zones (ridges). A wing surface representation of the four species under analysis from the watershed perspective is shown in Fig 3.

For better understanding of the application of the watershed transform in this work, lets consider a set {*m*_1_, *m*_2_, …, *m*_*k*_}, denoting the (*x*, *y*) coordinates of the points in the regional minima of the wing gradient image *g*(*x*, *y*) and *k* the number of detected particles. Hence, *P*(*m*_*k*_) is the set of points of the *k*^*th*^− particle, forming a connected component with the minimum point *m*_*k*_. Additionally, let *T*[*n*] be the set of points of *g*(*x*, *y*), lying below the geometrical plane *g*(*x*, *y*) = *n*; where *n* is an integer flood increment, which varies in the interval of [*min* + 1 ≤ *n* ≤ *max* + 1]. The *min* and *max* are the minimum and maximum intensity values of *g*(*x*, *y*), which can be determined from the histogram of *g*(*x*, *y*).

The algorithm slowly floods the surface of *g*(*x*, *y*) into a water bath at each step *n* and controls the number of points below the flood depth (points in *T*[*n*]). Thus, the set of points *P*_*n*_(*m*_*k*_) of the particle with the minimum *m*_*k*_ at stage *n* may be viewed as a binary image given by:

$$\begin{array}{c} P_{n}(m_{k})=P(m_{k})⋂T[n] \end{array}$$

where the value of the pixel at location (*x*, *y*) is constrained to:

$$\begin{array}{c} P_{n}(m_{k})=\{\begin{array}{cc} 1 & \;\text{if}\;\left(x,y\right)\in P\left(m_{k}\right)\;\text{and}\;\left(x,y\right)\in T\left[n\right]\\ 0 & \;\text{otherwise} \end{array} \end{array}$$

Eventually, the flooding process will reach the stage *n* = *max* + 1, and the union of all flooded basins (particles) is defined by:

$$\begin{array}{c} P[max+1]=\overset{k}{\underset{i=1}{⋃}}C(m_{i}) \end{array}$$

The standard algorithm does not allow mixing basins. Thereby it builds dams at the points of the first contact. These dams are considered the watershed lines and also the boundaries of image objects. In our case, we allow the algorithm to mix particle regions (basins with similar flood depth), maximizing the area of segmented zones (see Fig 1, step 2).

#### Feature calculation

A total of seven morphological features were used to classify the four biting midges species considered in this work (see Fig 1, step 3). Six features were computed from the binary image that contains the segmented particles of interest, including the number of particles (*F*_1_), and the mean values of the elongation (*F*_3_), solidity (*F*_4_), circularity (*F*_5_), hydraulic radius (*F*_6_), and eccentricity (*F*_7_). One feature, the total number of zones (*F*_2_), was computed from the binary image containing the segmented zones of interest. All values were normalized using the min-max method to bring them into the range from 0 to 1. The description and formulation of each of the computed features are shown in Table 1.

**Table 1:** Summary of computed features.

Feature	Description
*F*_1_ = *P*	*P* is the number of segmented particles.
*F*_2_ = *Z*	*Z* is the number of segmented zones.
$F_{3}=\frac{\sum_{i=1}^{P}\frac{m_{i}}{M_{i}}}{P}$	*m*_*i*_ is the minor axis of the *i*^*th*^−ellipse *M*_*i*_ is the major axis of the *i*^*th*^−ellipse.
$F_{4}=\frac{\sum_{i=1}^{P}\frac{Area_{i}}{ConvexArea_{i}}}{P}$	*Area*_*i*_ is the sum of all pixels in the *i*^*th*^−particle *ConvexArea*_*i*_ is the convex hull area of the set of points defining the *i*^*th*^−particle contour.
$F_{5}=\frac{\sum_{i=1}^{P}4\pi \frac{Area_{i}}{Perimeter_{i}^{2}}}{P}$	*Area*_*i*_ is the sum of all pixels in the *i*^*th*^−particle. *Perimeter*_*i*_ is the sum of all pixels in the *i*^*th*^−particle contour.
$F_{6}=\frac{\sum_{i=1}^{P}\frac{Area_{i}}{Perimeter_{i}}}{P}$	*Area*_*i*_ is the sum of all pixels in the *i*^*th*^−particle. *Perimeter*_*i*_ is the sum of all pixels in the *i*^*th*^−particle contour.
$F_{7}=\frac{\sum_{i=1}^{P}\frac{d_{i}}{M_{i}}}{P}$	*d*_*i*_ is the *i*^*th*^−ellipse foci. *M*_*i*_ is its major axis length.

#### MLC models

The separation between *Culicoides* classes on each dataset, for example, between *C. pusillus* and *C. obsoletus* or between *C. insignis* and *C. foxi* species can be viewed as a binary supervised learning problem that can be solved by training any MLCs with several input-output valid pairs of both biting midges species.

In this work, we considered the use of five MLCs belonging to the entropy and distance-based categories to find a decision boundary between biting midges classes (see Fig 1, step 4). According to the number of samples per species, the selected MLCs could provide reasonable classification performance without incurring the statistical assumption of not having the minimum required instances per sample or any overfitting during the models training process such as on artificial neural networks. A brief description of the employed classification algorithms are presented below:

- The naive Bayes (NB) classifier is based on probabilistic models with strong (naive) independence assumptions [32]. After training the NB classifier by estimating the class priors and the probability distribution of features, any test sample will follow the decision rule that provides the most probable value of the maximum *a posteriori* of the model.
- The support vector machine (SVM) classifier is based on the definition of an optimal hyperplane that linearly separates the training data [33]. This classifier aims to minimize the empirical risk and maximize the geometric maximum margin of the data points from the corresponding linear decision boundary.
- The k-nearest neighbors (kNN) classifier is a lazy learning (non-parametric) technique that invests a little effort into building the classifier and most of the work is performed at the time of classification [34]. It assigns a test sample to the predominating class in the voting scheme, which computed the euclidean distance between the test sample and each of the *k* = *k*_1_ + *k*_2_ + ⋯ + *k*_*n*_ neighbors (being *n* the number of neighbors to be considered for voting).
- The linear discriminant analysis (LDA) is a traditional classification model based on the calculation of the optimal data projection [35]. It minimizes the distances between instances of the same class while it maximizes the distance between instances of different classes. For a binary classification, observations are classified using the linear function: $g_{i}\left(x\right)=W_{i}^{T}x-c_{i}$, where *i* is the total number of classes, thus *i*: 1…2, $W_{i}^{T}$ is the transpose of a coefficient vector, *x* is a feature vector and *c*_*i*_ is a threshold. The model with the smallest value of *g*_*i*_(*x*) in the training becomes the best model to classify new instances.
- The random forest (RF) is an ensemble algorithm based on the combination of several empirical selected tree predictors and an average function for deciding the final instance classification [36].

The implementation of the proposed method was made in Matlab language [37], while the source code of all employed MLCs is available with the WEKA data mining software, version 3.6 [38].

### Experimental setup

This section outlines the experimental evaluation of the proposed method in terms of the detection and segmentation of particles within a wing, features calculation from segmented particles, and species classification. Also, we included a further feature relevance analysis to determine the features that most influence the *Culicoides* species separation.

#### Experimental dataset creation

As previously mentioned, two gnats databases were used as inputs to the proposed method to form two experimental datasets of feature vectors. In this setting, for each *Culicoides* wing image, a feature vector (i.e. a vector consisting of multiple elements) was obtained containing seven morphological features that represent the numerical description of the wing’s relevant regions. All feature vectors depicting satisfactory cases (i.e. well-segmented particles) were employed to create two experimental datasets serving to perform the feature relevance analysis and the biting midges classification. Thus, the fDataset, contains feature vector sets from the fDatabase, while the eDataset contains the corresponding feature vector sets obtained from the eDatabase.

#### MLC models optimization

The majority of MLCs were used in conjunction with the 10-cross validation method [39] in the training step to optimize the hyper-parameter tuning. Except for the NB classifier, which does not need to be configured, the SVM classifier used the regularization parameter (cost) varying from *c* = 10^−3^ to 10^3^, with an interval increment of 10 units and a linear kernel function. The kNN classifier included the estimation of the optimal value of *k*. It was optimized in the range from *k* = 1 to 20, and the contribution of each neighbor was weighted using the Euclidean distance to the instance to be classified. The LDA classifier, on the other hand, determined the coefficient vector $W_{i}^{T}$ with a constant value of *c*_*i*_ = 10^−6^ (small values are preferred), which is the ridge estimator used to guard against overfitting by penalizing large coefficients. While the RF classifier was optimized using the number of predictors from 100 to 1000 trees, with an increment of 100 units. Each tree used *log*_2_(*X*) + 1 for randomly attributes selection, being *X* the total number of attributes available in the current dataset.

#### Validation metrics

Regarding the classification stage, for all the MLCs used, the mean of the area under the curve of the receiver operating characteristic (AUC) was calculated to validate their performance. In addition, a statistical comparison between classifiers was performed using a paired-samples t-test with *α* = 0.05 [40]. This test allowed us to determine if there is a statistically significant difference between the classification models and, thus, select the most appropriate MLC to classify the biting midges species.

Additionally, we calculated the mean of the accuracy (ACC) metric to validate the detection of wing particles, the segmentation of the regions of interest using the proposed method, and to compare performance with previously developed methods. The error rate metric was also calculated to support the discussion of the limitation of the proposed method. Here, the error rate score is the complement of the successful performance in terms of the mean ACC, which is (100 − *ACC*)%.

## Results and discussion

A total of 192 biting midges wing images from two different databases (fDatabase and eDatabase) were used to feed the proposed method. The obtained results demonstrated quality performance in terms of wing particle detection and segmentation, of feature calculation, and of the *Culicoides* classification, as described next.

### Performance of the proposed method

**Fig 4:**
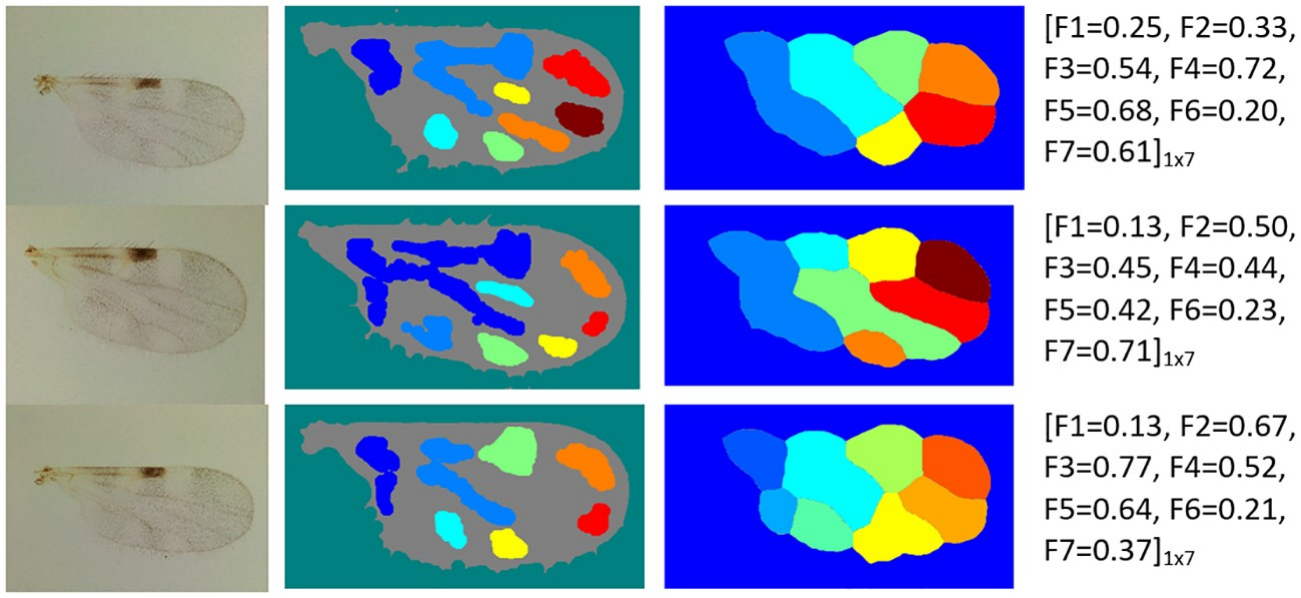
Performance of the proposed method on *C. pusillus* species samples. From left to right column: original wing image, particle detection, segmentation of zones of interest through the watershed method, and the final feature vector output obtained.

**Fig 5:**
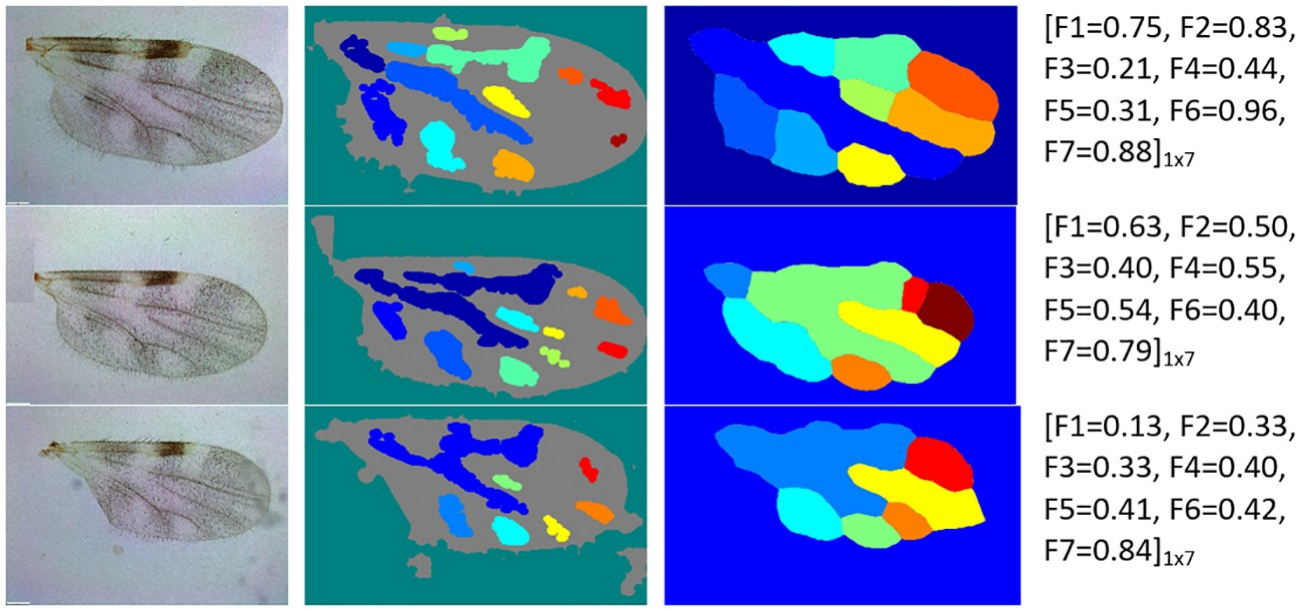
Performance of the proposed method on *C. obsoletus* species samples. From left to right column: original wing image, particle detection, segmentation of zones of interest through the watershed method, and the final feature vector output obtained.

**Fig 6:**
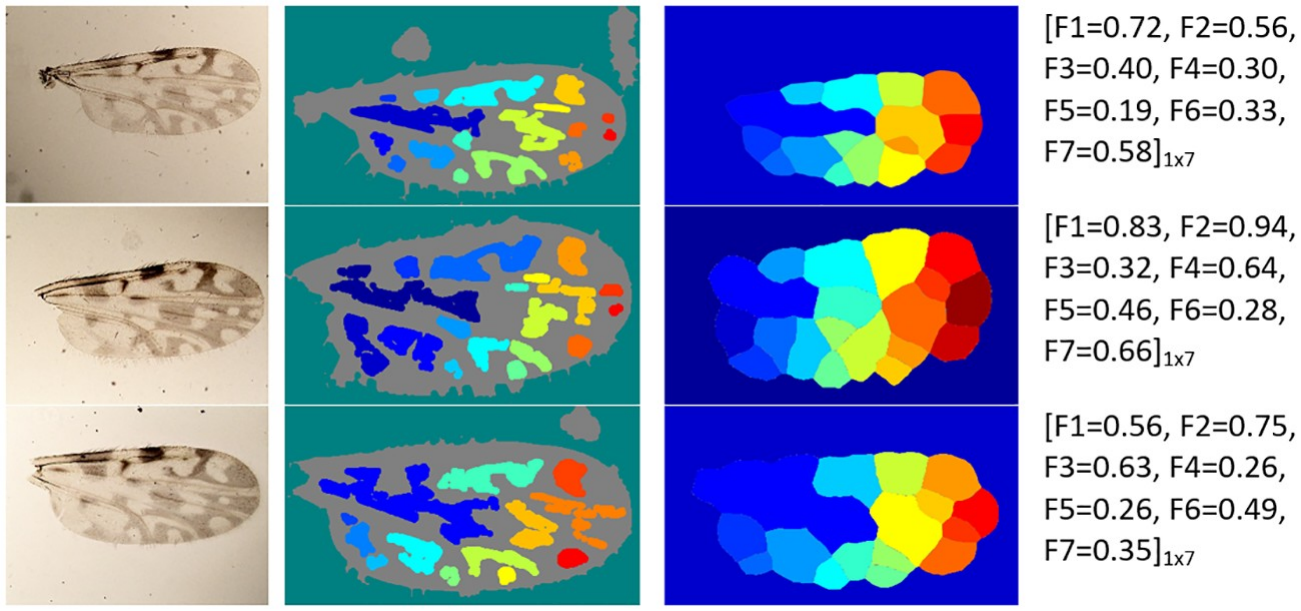
Performance of the proposed method on *C. foxi* species samples. From left to right column: original wing image, particle detection, segmentation of zones of interest through the watershed method, and the final feature vector output obtained.

**Fig 7:**
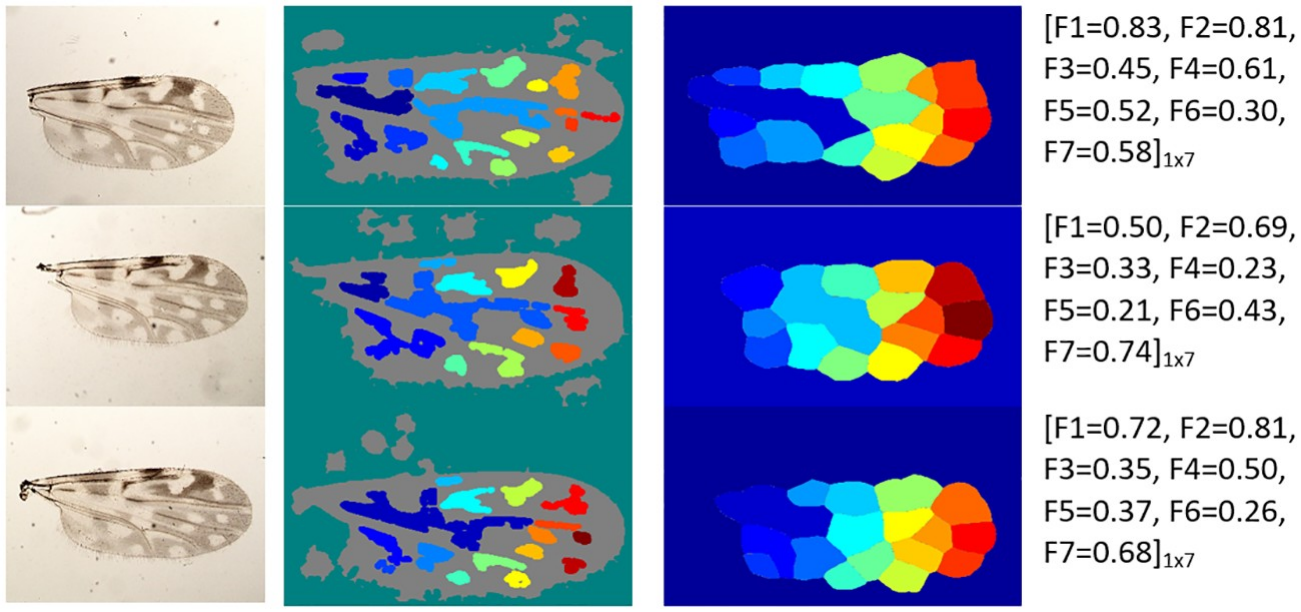
Performance of the proposed method on *C. insignis* species samples. From left to right column: original wing image, particle detection, segmentation of zones of interest through the watershed method, and the final feature vector output obtained.

In most cases, the proposed method achieved successful wing particle detection and region segmentation on the wing images. It reached mean ACC values of 96% and 84% on the fDatabase and the eDtabase, respectively. Some examples of the satisfactory image processing performance can be seen in Figs 4 and 5 for the fDatabase, and in Figs 6 and 7 for the eDatabase.

From these figures, it is possible to observe the main results related to the internal stages of the proposed method. The particle detection step (second column) correctly identified the stains (white areas) existing in the wing image. Similarly, the internal zones of the wing (third column) were correctly segmented. In both cases, the implemented multi-step image processing algorithm facilitated the tasks and it improved the quality of the wing image to highlight the stains and boundary details. It provided the binarized image related to the wing that leads the watershed transform to a successful determination of the basins and ridges. In this way, the better the basins estimation, the better the stains segmentation. Ridges are linked to irrelevant wing image areas.

It also should be pointed out that there are wing morphological changes among considered biting midges species that are possible to track when analyzing the wing particle detection resultant images (second column in Figs 4 to 7). The most noticeable aspect is related to the number of particles presented on each species. For example, the *C. pusillus* and *C. obsoletus* species provided a lesser number of particles when compared to the *C. insignis* and *C. foxi* species. Also, the particle size and shape offered singular variation among species. Particles in the *C. pusillus* and *C. obsoletus* samples are bigger and tend to a rounded shape with regular contour. Meanwhile, particles for the *C. insignis* and *C. foxi* samples are smaller with irregular contour and shapes. Similarly, when analyzing the zone segmentation in the resultant images (third column in Figs 4 to 7), it is possible to observe the same morphological difference among biting midges species. Samples of *C. pusillus* and *C. obsoletus* showed a lower number of zones with larger size respect to the *C. insignis* and *C. foxi* species. This effect was expected, since the number of zones have a strong connection to the number of particles inside the wing.

#### Limitations

**Fig 8:**
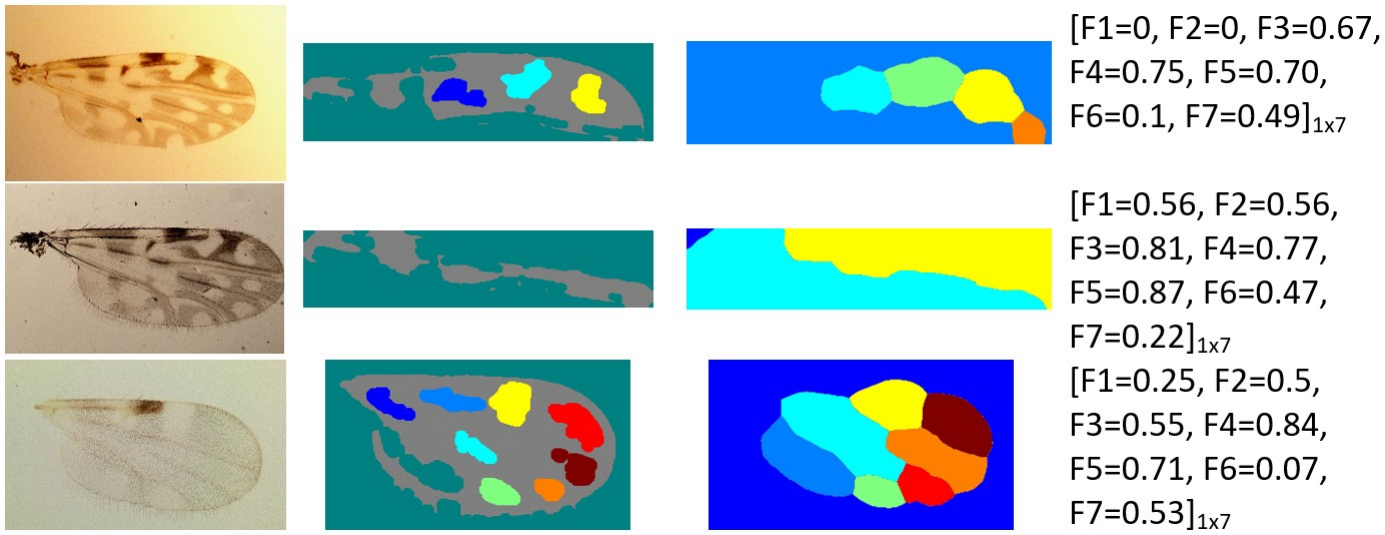
Examples of unsuccessful performance of the proposed method on different species samples. From left to right column: original wing image, particle detection, segmentation of zones of interest through the watershed method, and the final feature vector output obtained.

Despite the good obtained results, there was an overall error value of 4% and 16% for the fDatabase and eDatabase, respectively. These error rates are due to the failure in the wing particle determination and segmentation step of the proposed method. Fig 8 shows an example of the effect of a wrong wing detection, where the bounding box within the wing image is out of the region of interest, and thus, the watershed method spreads out of the box, leading to reduced performance. This situation is related to the wing image acquisition protocol used in the laboratory, for example, an improper illumination condition when capturing the image (Fig 8 first row), the wing size (magnified by the lens), making the wing border to touch the outer limit of the region of interest (Fig 8 second row), and the intensity of the pixels located in the wing contour similar to the image background (Fig 8 third row), which could affect the performance of the proposed method.

#### Feature calculation performance

Regarding feature calculation, the wing morphological changes were also expressed in terms of numerical values computed from the segmented particles and zones within the wing (fourth column in Figs 4–7). The mean of the number of particles (*F*_1_ feature) are very similar for the *C. pusillus* and *C. obsoletus* species, reaching values of 0.34 and 0.39, respectively. Similarly, the mean of the number of zones (*F*_2_ feature) exhibited almost the same behavior between both species, attaining values of 0.57 and 0.55 for the *C. pusillus* and *C. obsoletus*, respectively. It should be pointed out that both *F*_1_ and *F*_2_ features are related.

Other features, such as the mean of the elongation (*F*_3_) obtained from the particles of *C. pusillus* samples, tend to be less elliptical than those from *C. obsoletus* samples. These results are corroborated by the reached values of elongation of 0.66 for the *C. pusillus* versus the 0.35 for the *C. obsoletus*. The mean of the solidity (*F*_4_) feature, on the other hand, is related to the shape of the particles, and the attained values of 0.64 against 0.38 explains the reason why particles of the *C. pusillus* species exhibited more regular shape contours to the *C. obsoletus* species. The mean of the circularity (*F*_5_) feature achieved values of 0.68 and 0.36 for the *C. pusillus* and *C. obsoletus* species, respectively, which means that the shape of the particles of the *C. pusillus* species are more circular than particles from the *C. obsoletus* species. These results are very similar to those obtained with the mean of the elongation feature (*F*_3_). However, the *F*_3_ is focused on the relationship between the major and minor elliptical axis, while the *F*_5_ involves the area, perimeter, and radial based quadrants. The mean of the hydraulic radius (*F*_6_) feature is a function used to describe the shape of the particle as if it were a circle and how efficient its perimeter is in relation to the area contained by it. The obtained values for this feature were 0.30 and 0.70 for the *C. pusillus* and *C. obsoletus* species, respectively. The mean of the eccentricity (*F*_7_) feature defines how far from being circular the particle is, values close to zero means the particle shape is more circular and vice-versa. In this setting, the values of 0.41 and 0.76 were obtained for the *C. pusillus* and *C. obsoletus* species, respectively. These results demonstrated that the wings of *C. obsoletus* contained particles far from being circular.

Meanwhile, when comparing the feature values between the *C. foxi* (Fig 6) and *C. insignis* (Fig 7) species belonging to eDatabase, this reveals that the *C. foxi* species has a higher number of particles (*F*_1_) than the *C. insignis*, with a mean value of 0.73 and 0.51, respectively. Taking into account that the number of particles (*F*_1_) feature is related to the number of zones (*F*_2_) feature (third column in Figs 6 and 7), it is to be expected that the *C. foxi* species will show more zones with a mean value of 0.74 versus the mean value of 0.64 for the *C. insignis* species. In contrast with the species from the fDatabase, for the species in the eDatabase, the first two features seem to be more relevant for distinguishing between the species analyzed.

Regarding the morphology variation of the wing particles (second column in Figs 6 and 7), the analysis of the mean of the elongation (*F*_3_) feature shows that the particles of the *C. foxi* species tend to be more elliptical than the ones of the *C. insignis*, with values of 0.43 and 0.56, respectively. The mean value of the solidity (*F*_4_) feature for *C. foxi* species is 0.62 and for the *C. insignis* is 0.55, which means that the particles of the *C. foxi* wing samples have a slightly more regular shape than those ones of *C. insignis*. The mean of the circularity (*F*_5_) feature reveals that the particles of *C. insignis* wings have a shape more similar to a circle than the particles of *C. foxi* wings, with values of 0.54 and 0.44, respectively. The mean of the hydraulic radius (*F*_6_) feature, on the other hand, points out that *C. insignis* species have particles with a greater area ratio inside the perimeter than *C. foxi*, with values of 0.42 and 0.36, respectively. Finally, the mean of the eccentricity (*F*_7_) feature exhibits that the particles of the *C. foxi* are less similar to a circle than the particles of *C. insignis*, with values of 0.53 and 0.45, respectively.

### Feature relevance

We applied an all-versus-all strategy on the features space to find the most relevant features for separating the biting midges classes in the fDataset, eDataset, and both datasets together. The procedure was carried out by feeding the selected MLCs with all the feature combinations. For the selection criteria of relevance, we follow two general rules: (1) from each classifier, the model that produces the highest AUC score was selected to determine the best subset of features; else (2) if there is a model that did not provide AUC-based statistical difference with respect to the best model selected in the previous step, the minimal subset (optimal) of features was preferred. Due to the few amount of computed features, this strategy was easily performed, and the obtained results are shown in Table 2.

**Table 2:** Summary of the statistical comparison based on the AUC performance between the best and optimal feature subset per classifier.

Dataset	Best model	Best feature subset	AUC±SD	Optimal model	Optimal feature subset	AUC±SD	t-test (*α* = 0.05)
fDataset	NB	*F*_2_, *F*_5_, *F*_6_, *F*_7_	0.99 ± 0.03	NB	*F*_6_, *F*_7_	0.98 ± 0.05	*p* = 0.06
	SVM (c = 1000)	*F*_1_, *F*_2_, *F*_5_, *F*_6_, *F*_7_	0.99 ± 0.04	SVM (c = 100)	*F*_3_, *F*_5_, *F*_6_, *F*_7_	0.97 ± 0.06	*p* = 0.08
	kNN (k = 1)	*F*_1_, *F*_3_, *F*_4_, *F*_6_, *F*_7_	1 ± 0.01	kNN (k = 15)	*F*_6_, *F*_7_	0.98 ± 0.05	*p* = 0.42
	LDA	*F*_3_, *F*_4_, *F*_6_, *F*_7_	1 ± 0.01	LDA	*F*_6_, *F*_7_	0.98 ± 0.05	*p* = 0.05
	RF (i = 100)	*F*_4_, *F*_6_, *F*_7_	0.98 ± 0.04	RF (i = 100)	*F*_6_, *F*_7_	0.96 ± 0.05	*p* = 0.07
5*eDataset	NB	*F*_1_, *F*_5_	0.87 ± 0.12	NB	*F*_1_	0.86 ± 0.13	*p* = 0.08
	SVM (c = 10)	*F*_1_, *F*_4_	0.83 ± 0.13	SVM (c = 10)	*F*_1_, *F*_4_	0.83 ± 0.13	*p* = 1.00
	kNN (k = 15)	*F*_1_	0.87 ± 0.13	kNN (k = 15)	*F*_1_	0.87 ± 0.13	*p* = 1.00
	LDA	*F*_1_, *F*_4_, *F*_5_, *F*_6_	0.91 ± 0.11	LDA	*F*_1_, *F*_6_	0.90 ± 0.12	*p* = 0.37
	RF (i = 200)	*F*_1_, *F*_3_	0.87 ± 0.13	RF (i = 200)	*F*_1_, *F*_3_	0.87 ± 0.13	*p* = 1.00
Both*	NB	*F*_1_, *F*_2_, *F*_5_	0.96 ± 0.03	NB	*F*_1_, *F*_2_, *F*_5_	0.96 ± 0.03	*p* = 1.00
	SVM (c = 10)	*F*_1_, *F*_2_, *F*_3_, *F*_4_, *F*_5_, *F*_6_, *F*_7_	0.92 ± 0.05	SVM (c = 10)	*F*_1_, *F*_2_, *F*_4_	0.90 ± 0.06	*p* = 0.07
	kNN (k = 15)	*F*_1_, *F*_2_, *F*_4_	0.96 ± 0.03	kNN (k = 15)	*F*_1_, *F*_2_, *F*_4_	0.96 ± 0.03	*p* = 1.00
	LDA	*F*_1_, *F*_4_, *F*_5_, *F*_6_	0.97 ± 0.03	LDA	*F*_1_, *F*_2_, *F*_5_	0.96 ± 0.03	*p* = 0.06
	RF (i = 100)	*F*_1_, *F*_2_, *F*_3_, *F*_6_, *F*_7_	0.96 ± 0.03	RF (i = 100)	*F*_1_, *F*_2_, *F*_3_	0.95 ± 0.04	*p* = 0.05

*fdataset plus eDataset; c-Cost; k-number of neighbors; i-number of trees; SD-standard deviation; F-feature.

For the fDataset, the best subset of features varied from three to five descriptors across the employed MLCs. The selected optimal subset of features was composed of only two descriptors, the mean of the hydraulic radius (*F*_6_) and eccentricity (*F*_7_). From Table 2, it is possible to read that there was almost a total consensus among all MLCs on deciding the optimal subset. This result is explained by the particularity of the features and the mean shape of the wing particles. The *F*_6_ measures the efficiency of hydraulic channel flow by considering the cross-sectional area and perimeter. In this work, this feature considered the mean of the area and perimeter of the wing particles. As the *C. pusillus* samples provided a greater mean area and perimeter of particles respect to the *C. obsoletus* samples, the difference values in this feature helps to separate both species. On the other hand, the *F*_7_ feature measures how closely a conic section resembles a circle. Although this feature provides values greater than 0.5, i.e., the average shape of the wing particles is elliptical for both species, the conical shape of particles in the *C. pusillus* samples were less elliptical than the *C. obsoletus* samples. This score difference supports the discrimination of *C. pusillus* and *C. obsoletus* species.

**Fig 9:**
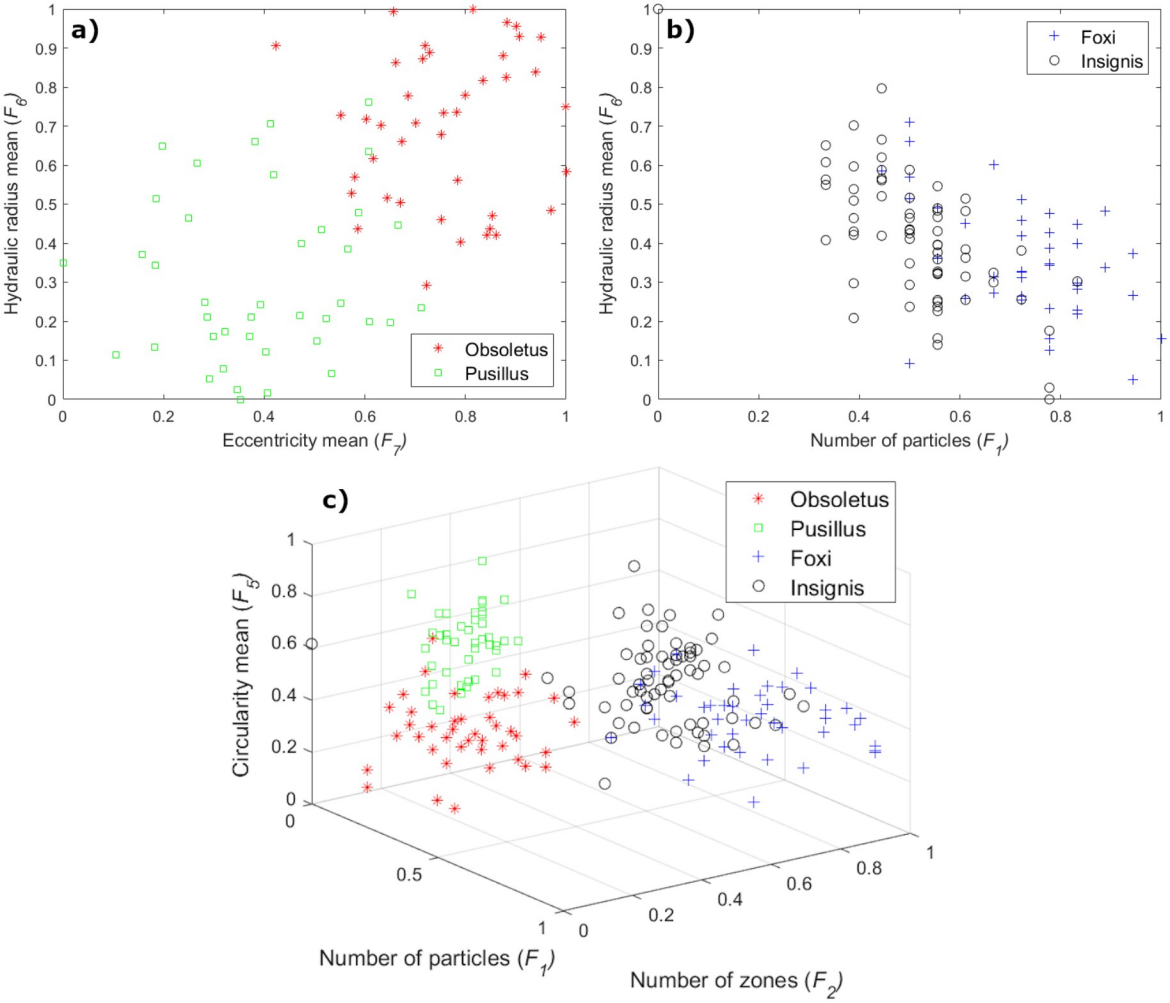
Biting midges species separation according to the optimal subset of features in (a) fDataset, (b) eDataset, and (c) both datasets together.

An example of computed feature vectors of both species is shown in Figs 4 and 5, fourth column. Also, a visual representation of how well the optimal subset of features separated both biting midges classes is shown in Fig 9a. As shown in this figure, the two species are in two cluster areas that can be easily separated using a straight line.

For the eDataset, unlike the fDataset, the best subset of features varied from one to four descriptors across the used MLCs. However, the selected optimal subset of features was formed by two descriptors, namely, the number of particles (*F*_1_) and the mean of the hydraulic radius (*F*_6_). From Table 2, it is possible to read that all MLCs included the *F*_1_ feature in the final subset, but only the LDA classifier considered the *F*_6_ feature, obtaining the highest AUC classification performance. Since there are morphological differences in terms of particles from species to species, the *F*_1_ feature appeared as an important descriptor to separate the *C. foxi* and *C. insignis* classes. Like in the case of the fDataset, the *F*_6_ feature discriminates both biting midges species, according to the mean of the area and perimeter of the wing particles.

An example of computed feature vectors of both species is shown in Figs 6 and 7, fourth column. Besides, a visual representation of how well the optimal subset of features separated the classes is presented in Fig 9b. From this figure, it should be noted that there is not a clear boundary between both species. Thus, the separation becomes more difficult using linear classifiers.

Concerning the classification of the four species in both datasets (fDataset plus eDataset), the best subset of features varied from three to seven descriptors among all MLCs, but the selected optimal subset of features involved the number of particles (*F*_1_), number of zones (*F*_2_), and the mean of the circularity (*F*_5_) features. From Table 2, it is possible to read that all MLCs considered the *F*_1_ and *F*_2_ features as relevant descriptors in the final subset. The *F*_5_ feature was selected by its frequency of participation in the best classification schemes, e.g., the LDA and NB classifiers decided for the *F*_5_ feature while the kNN considered the *F*_4_ feature. The union of both datasets represents a more complicated feature space than the single fDataset or eDataset spaces. Thus, features such as the *F*_1_ and *F*_2_ oriented to the particles within the wings are prone to gain more relevance due to the particular wing morphology of each species (different amount of particles per species). On the other hand, the relevance of the *F*_5_ feature is given by the wide variation in the mean of the circularity of the particles across the four species under analysis, which is well appreciated to separate these classes.

An example of how well the optimal subset of features separated the classes is presented in Fig 9c. From this figure, it is possible to observe that samples of each dataset tend to be clustered. However, the limits of the classes are well defined, minimizing the false-positive classification.

Overall, the AUC-based classification performance analysis of the seven features computed by the proposed method, when applied to the experimental datasets, highlighted that the relevance of the features points out to the morphology of the wing particles in the fDataset (*F*_6_ and *F*_7_), and to a balanced combination between the morphology of the wing particles and the number of particles in the eDataset (*F*_1_ and *F*_6_). However, when applied to both datasets, the relevance of features indicates an unbalanced combination among the morphology of the wing particles, and the number of particles and zones (*F*_1_, *F*_2_ and *F*_5_).

### Biting midges classification

Table 3 highlights the classification performance obtained by selected MLCs using the optimal subset of features per dataset. These results served to support the satisfactory performance provided by the proposed method in terms of feature calculation. As the previous section shows, the optimal subsets of features allow differentiating the classes by merely plotting the features and separate them by a line. This precedent suggests that the different MLCs applied have a high performance separating the species.

**Table 3:** Summary of the statistical comparison based on the AUC performance of the best classifiers per dataset.

Dataset	Highest performance model	AUC ± SD	Other model	AUC ± SD	t-test (*α* = 0.05)
fDataset	NB	0.98 ± 0.05	SVM (c = 100)	0.97 ± 0.06	*p* = 0.14
			kNN (k = 15)	0.98 ± 0.05	*p* = 0.09
			**LDA**	**0.98 ± 0.05**	***p* = 0.93**
			RF (i = 100)	0.96 ± 0.05	*p* < 0.05
eDataset	**LDA**	**0.90 ± 0.12**	NB	0.86 ± 0.13	*p* < 0.05
			SVM (c = 10)	0.83 ± 0.13	*p* < 0.05
			kNN (k = 15)	0.87 ± 0.13	*p* < 0.05
			RF (i = 200)	0.87 ± 0.13	*p* < 0.05
Both*	NB	0.96 ± 0.03	SVM (c = 10)	0.90 ± 0.06	*p* < 0.05
			kNN (k = 15)	0.96 ± 0.03	*p* = 0.65
			**LDA**	**0.96 ± 0.03**	***p* = 0.38**
			RF (i = 100)	0.95 ± 0.04	*p* = 0.09

*fdataset plus eDataset; c-Cost; k-number of neighbors; i-number of trees; SD-standard deviation; bold line means the selected model.

**Fig 10:**
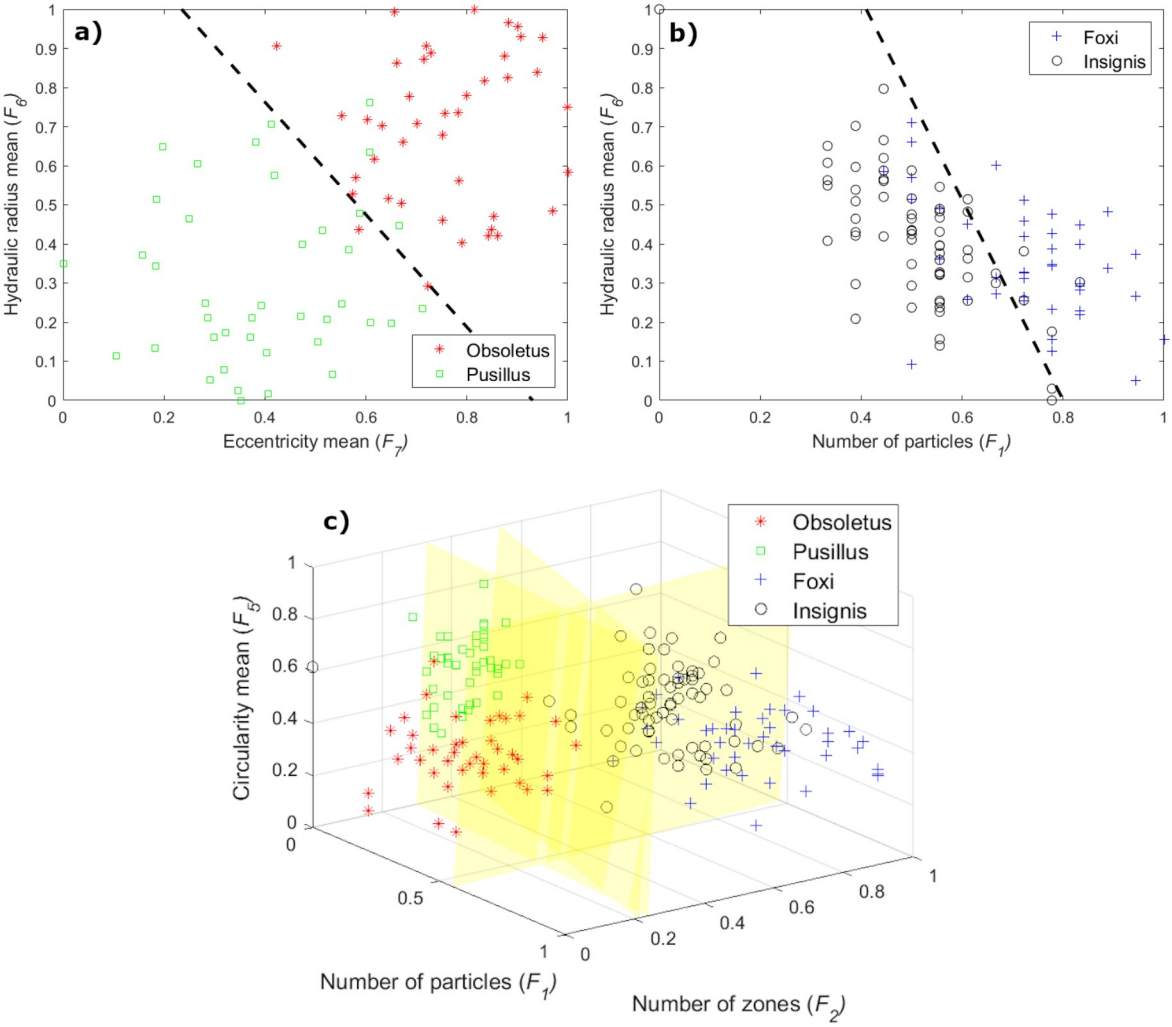
Biting midges species separation according to the LDA classifier using the optimal subset of features in (a) fDataset, (b) eDataset, and (c) both datasets together.

The results obtained by the proposed method applied to the fDataset reveals that the kNN (*k* = 15), NB, and LDA classifiers achieved the best classification performance, as highlighted in Table 3. The three classifiers obtained the same satisfactory performance without AUC-based statistical difference at *α* = 0.05, attaining a mean of AUC score of 0.98. However, the kNN exhaustively explored the total space of *k* values, demanding more iterations and memory use. The NB is centered on the entropy measure, and its performance is biased to those features that provide high variation, making it practical for specific situations. On the other hand, the LDA is benefited from features that provide samples’ separation linearly, avoiding incurring any bias. Thus, we selected the LDA as the best classifier to differentiate the species in this dataset. A visual representation of the LDA performance on the optimal feature space is shown in Fig 10a. As can be seen from this figure, the LDA classifier can distinguish both species with only a few samples mismatched.

For the eDataset, the proposed method reached the best results with the LDA classifier using the optimal subset of features, as shown in Table 3, accomplishing a mean of AUC score of 0.90. This result was statistically superior to the remaining models. The feature space in this dataset was more challenging than the one in the fDataset. Still, the LDA classifier successfully decides the area and defines the appropriate boundary for each species, as shown in Fig 10b.

Additionally, the scenario in which all four species are classified together was also studied, as described in Table 3. From this table, it is possible to read that the kNN (*k* = 15), NB, and LDA classifiers obtained the same satisfactory classification performance with the optimal subset of features, attaining a mean of AUC score of 0.96. These results did not represent a statistical difference (*α* = 0.05) in terms of AUC performance among them. However, likewise, in previous cases, we selected the LDA as the best classifier to differentiate the four species, as shown in Fig 10c, where it is possible to notice the separation hyperplanes established by the LDA classifier.

Further analysis of the results in this scenario is shown in the confusion matrix of Table 4. As it can be seen, the species from the fDataset obtained the highest true positive rate (98% for *C. obsoletus* and 95% for *C. pusillus*), while the species from the eDataset obtained lower values (81% for *C. foxi* and 85% for *C. insignis*). These results were expected, considering the results obtained by analyzing these datasets separately.

**Table 4:** Confusion matrix obtained by the LDA classifier for the four *Culicoides* species classification problem.

		Predicted Class			
		*C. obsoletus*	*C. pusillus*	*C. foxi*	*C. insignis*
True Class	*C. obsoletus*	41	1	0	0
	*C. pusillus*	2	40	0	0
	*C. foxi*	0	0	34	8
	*C. insignis*	1	0	9	56

#### Comparison with previously developed methods

We carried out a direct comparison against the image-enhancing methods previously developed by Guerrón et al. [23], and Benalcázar et al. [24], based on the wings and particles (inside the wing) segmentation performance. The three approaches used Otsu’s method [25] as part of the segmentation stage. Thus, we applied these approaches to both experimental databases (fDatabase and eDatabase). In this comparison, the ACC computation was made on two different scenarios. First, on the wing segmentation in the image sample. If the wing area is isolated (not connected) from non-desired objects in the background, then the segmentation result is positive. And second on the particle segmentation inside the wing. If all the particles are determined, and their contours are closed, the segmentation result is positive. The obtained ACC-based segmentation results are shown in Table 5.

**Table 5:** Wings and particles ACC-based segmentation performance for the four species.

Species	Guerrón et al. [23]		Benalcázar et al. [24]		Proposed method	
	ACC^±^	ACC*	ACC^±^	ACC*	ACC^±^	ACC*
*C. obsoletus*	7.14	9.52	90.48	88.10	100	100
*C. pusillus*	0	0	80.95	66.67	100	100
*C. foxi*	4.76	11.90	85.71	80.95	95.24	97.62
*C. insignis*	0	7.58	46.97	57.58	89.39	86.46
**Total**	2.60	7.29	72.40	71.35	**95.31**	**94.79**

^±^accuracy in wings segmentation;

*accuracy in particles segmentation; All values are in percent.

From this table, it is possible to read that the proposed method was the best for both segmentation aspects (wings and particles) for all species, obtaining total ACC scores of 95.31% and 94.79%, respectively. It was followed by the method proposed in [24], which obtained ACC scores of 72.40% and 71.35%. Finally, the approach proposed in [23] provided the worst performance, attaining a total of ACC score of 2.60% and 7.29%. Besides, it should be noted that *C. obsoletus* and *C. pusillus* species provided an easier scenario for the proposed method. In contrast, the *C. obsoletus* and *C. foxi* species were better scenarios for methods proposed in [24] and [23]. The *C. insignis* species was the toughest scenario, as it achieved the lowest performance for each method.

These results are related to the preprocessing scheme used by each method before applying the segmentation step. The method proposed in [23] only used a Gaussian filter with a size 3 and *σ* = 0.5 to prepare the image. The use of this filter removes some noises, but it also blurs the edges and reduces contrast, making the segmentation task harder. The approach described in [24] enhanced the method proposed in [23] by substituting the Gaussian filter with a (5 × 5) median filter, improving the denoising part while conserving the edges detail. Then, a histogram equalization operation is also performed to enhance the contrast of the image. These improvements to the method proposed in [24] could be enough for carrying out the wing segmentation, but they are insufficient for segmenting the particles inside the wing. Either way, this method performs better than the one described in [23].

On the other hand, in the proposed method, a median filter with a kernel size of (15 × 15) was included to remove noise. At the same time, the wing and particle contours remain intact as much as possible. Then, adaptive histogram equalization is applied to enhance the contrast of the image per image zones. Subsequently, a Wiener filter is used with a kernel size of (25 × 25) to remove any noise that remains after the median filter applied in the previous step while the wing and particle contours are preserved. This enhanced scheme makes the proposed method more robust in terms of image preprocessing. Thus, the obtained segmentation results were somewhat expected.

**Fig 11:**
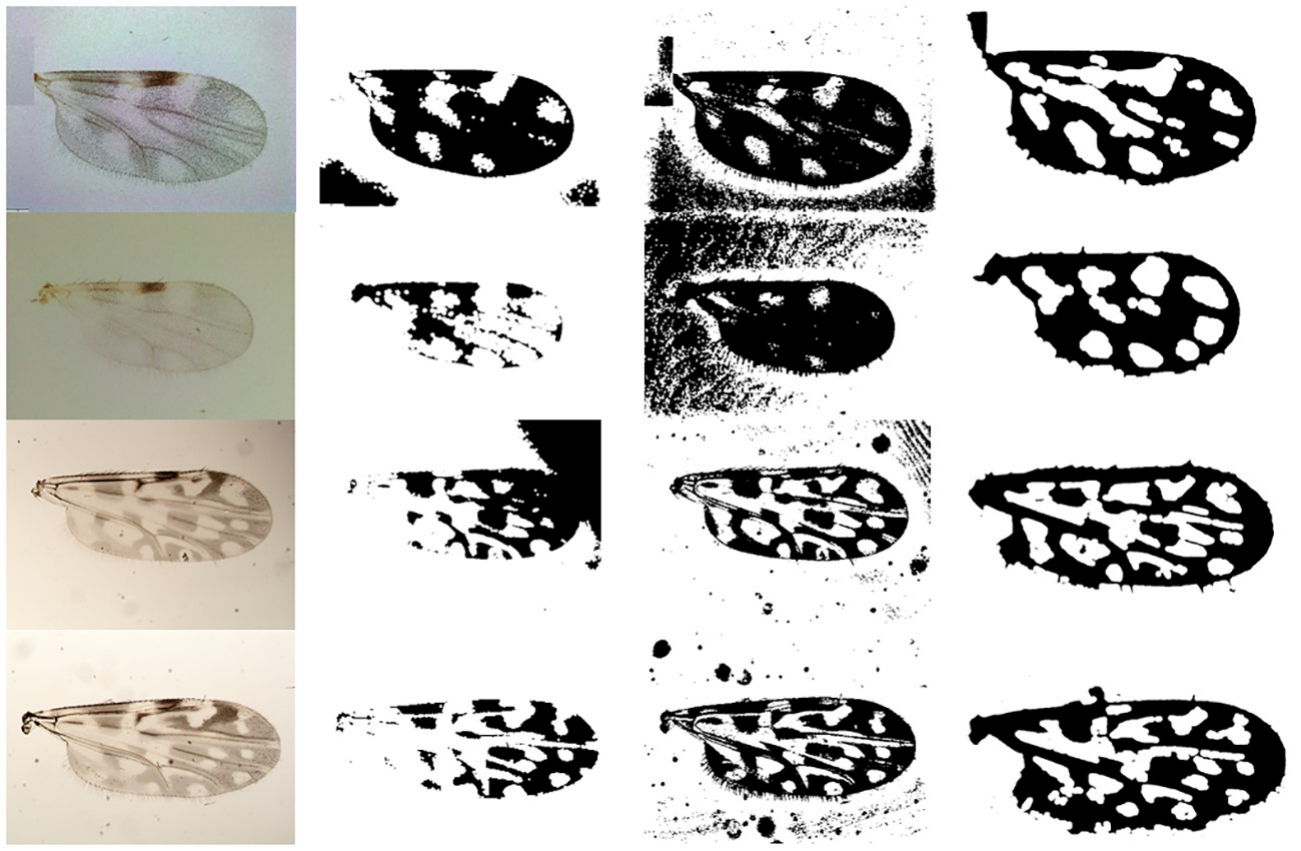
Wing and particles segmentation performance using the Otsu’s method on *C.obsoletus* (first row), *C.pusillus* (second row), *C.foxi* (third row), and *C.insignis* (fourth row) samples. From left to right, original wing image, Guerrón et al. [23], Benalcázar et al. [24], and proposed method results.

An example of each method segmentation performance on a random sample from each species is shown in Fig 11. As can be seen, the limited performance of the method proposed in [23] for all samples. Only the *C. obsoletus* sample (first row) was segmented correctly, but the particles inside the wing in all samples were not. The method proposed in [24] is able to segment all the wings successfully, however, it fails in segmenting the particles inside the wing of two samples (see Fig 11, first and second rows). In contrast, the proposed method demonstrated to be satisfactory for segmenting both the wing and particles on all the test samples.

**Fig 12:**
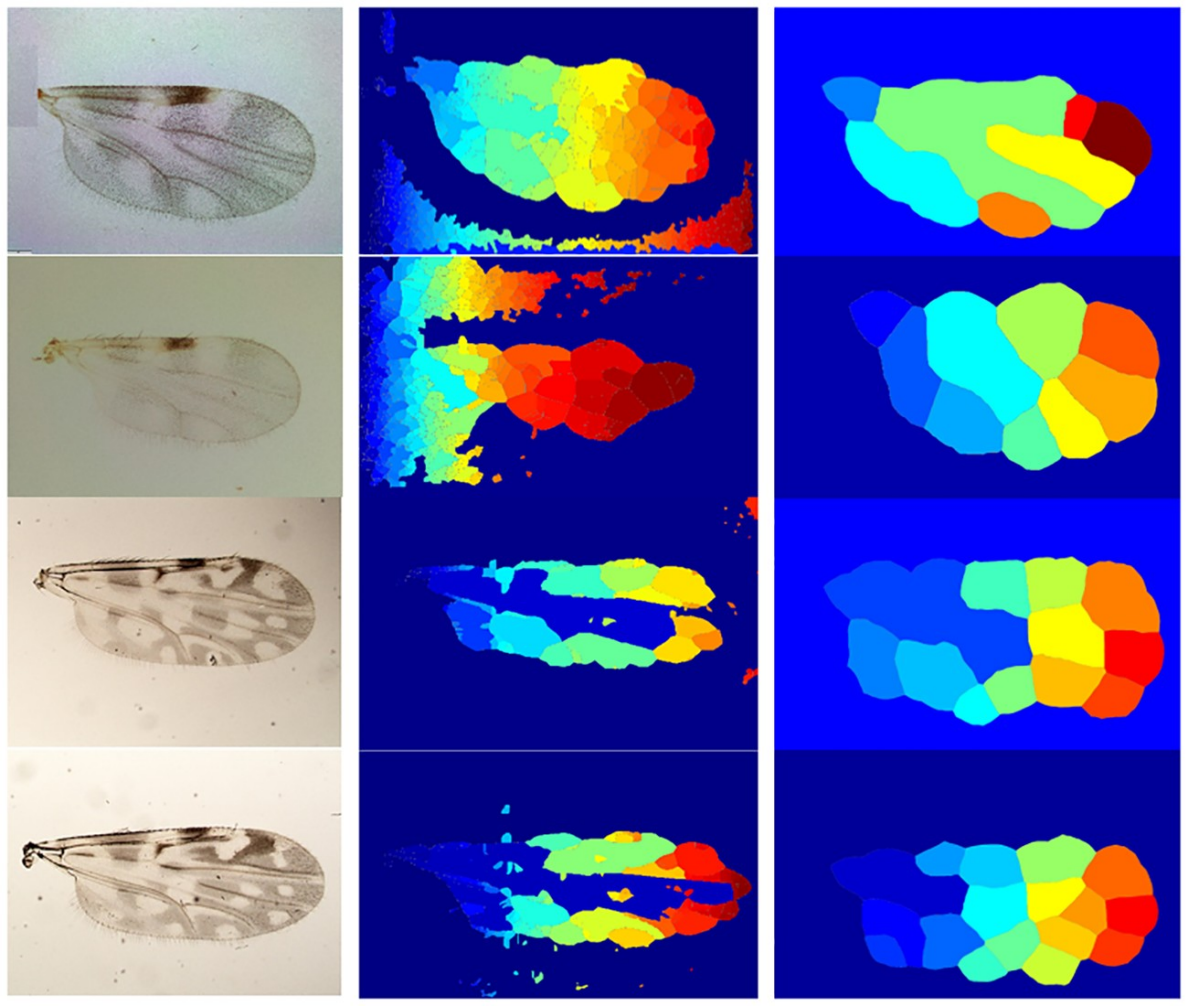
Resulting segmented zones obtained by the watershed method on *C.obsoletus* (first row), *C.pusillus* (second row), *C.foxi* (third row), and *C.insignis* (fourth row) samples. Left: original wing image. Center: results obtained after applying the method proposed by Benalcázar et al. [24]. Right: results obtained after applying the method proposed in this paper.

Moreover, having a proper segmentation of the particles inside the wings can be noticed in Fig 12, where the watershed performance obtained by the approach of Benalcázar et al. [24] (second column) and the proposed method (third column), are shown. From this figure, it is possible to conclude that an unsuccessful wing or particles segmentation makes the watershed method to overflow, thus, provoking wrong calculation of features that depend on the segmentation process, e.g., the number of zones (*F*_2_) is calculated from the particles inside the wing.

In general, these results show the importance of selecting an appropriate preprocessing scheme to improve the segmentation quality and the further feature calculation and classification stages. Although previous works demonstrated good performance in some cases, the approach proposed in this work yielded better performance and accuracy. Therefore, the results obtained are promising, indicating that the proposed method could be extended to analyze other species, and good results could also be expected.

## Conclusion

We proposed a two-stage method for analyzing four biting midges species, namely, *C. obsoletus*, *C. pusillus*, *C. foxi* and *C. insignis*. First, the image processing task improved the image quality to fulfill a segmentation of particles of interests, and then, the segmentation of the zones of interests inside the biting midge wing was made by the watershed transform. The proposed method was able to produce optimal features vectors that help to identify the biting midges species. Feature relevance analysis indicated that the mean of hydraulic radius and eccentricity were relevant to find the decision boundary between *C. obsoletus* and *C. pusillus* species. The number of particles and the mean of the hydraulic radius for deciding between *C. foxi* and *C. insignis* species, while the number of particles and zones, and the mean of circularity for distinguishing among the four species. The LDA classifier emerged as the best model for fDataset, eDataset, and both datasets together, reaching mean AUC scores of 0.98, 0.90, and 0.96, respectively.

Future works include experimentation with larger image datasets, and to include other species of *Culicoides* such as species of subgenera *Avaritia*, *Haematomyidium*, *Hoffmania* or *Oecata*. Further enrichment of the dataset would allow for an increase in species identification accuracy, improving our proposed method as a tool for entomological surveillance and distinguishing between vector and non-vector species. We also want to explore the use of deep learning techniques and the implementation of an embedded solution in a mobile device.
